# Genomic diversity and versatility of *Lactobacillus plantarum*, a natural metabolic engineer

**DOI:** 10.1186/1475-2859-10-S1-S3

**Published:** 2011-08-30

**Authors:** Roland J Siezen, Johan ET van Hylckama Vlieg

**Affiliations:** 1 Kluyver Centre for Genomics of Industrial Fermentation, NIZO food research, P.O. Box 20, 6710 BA Ede, The Netherlands; 2 Centre for Molecular and Biomolecular Informatics, NCMLS, Radboud University Medical Centre, PO Box 9101, Nijmegen, the Netherlands; 3 TI Food and Nutrition, P.O. Box 557, 6700 AN Wageningen, The Netherlands; 4 Netherlands Bioinformatics Centre, 260 NBIC, P.O. Box 9101, 6500 HB Nijmegen, the Netherlands; 5 Danone Research, R.D. 128, Avenue de la Vauve, F-91767 Palaiseau Cedex, France

## Abstract

In the past decade it has become clear that the lactic acid bacterium *Lactobacillus plantarum* occupies a diverse range of environmental niches and has an enormous diversity in phenotypic properties, metabolic capacity and industrial applications. In this review, we describe how genome sequencing, comparative genome hybridization and comparative genomics has provided insight into the underlying genomic diversity and versatility of *L. plantarum*. One of the main features appears to be genomic life-style islands consisting of numerous functional gene cassettes, in particular for carbohydrates utilization, which can be acquired, shuffled, substituted or deleted in response to niche requirements. In this sense, *L. plantarum* can be considered a “natural metabolic engineer”.

## Background

The lactobacilli constitute a major group of the Lactic Acid Bacteria (LAB). They occupy a wide range of niches and are generally found in environments with high levels of carbohydrates, such as food products (dairy products, fermented meat, sourdoughs) as well as (fermenting) plant-derived substrates. In addition, they occupy different niches on and in the human body including the respiratory, gastrointestinal and urogenital tract. As a consequence, lactobacilli have been studied extensively, initially mainly because of their importance for food production. More recently, there is a rapid increase in literature focusing on their occurrence and activity in the human microbiota as well as their use as probiotics, defined as ‘‘live microorganisms which when administered in adequate amounts confer a health benefit on the host’’ (http://www.who.int).

The genus *Lactobacillus* belongs to the phylum Firmicutes, class Bacilli, order Lactobacillales, and family Lactobacillaceae. A comprehensive review of the taxonomy of lactobacilli summarizes the current taxonomic as well as historic changes [1]. As for many other genera, the taxonomy of lactobacilli has been subject to several changes since the emergence of molecular technologies. At the moment of writing of this review (April 15, 2011), 173 species are recognized, and after removing synonymous species names due to reclassification this can be reduced to 141 species. http://www.bacterio.cict.fr/l/lactobacillus.html.

Many *Lactobacillus* species are highly specialized and are only found in a limited number of niches. A well-known example is the species *Lactobacillus delbrueckii*, which is highly adapted to the dairy environment and widely applied in yoghurt manufacture. Other species, such as *Lactobacillus acidophilus*, *Lactobacillus johnsonii*, *Lactobacillus reuteri* and *Lactobacillus rhamnosus* are typical inhabitants of the GI tract, and are used in probiotic products [2]. The genome sequence of *Lactobacillus iners*, a predominant member of the vaginal microbiota, was recently shown to have undergone extensive gene loss, resulting in the smallest *Lactobacillus* genome reported to date [3].

In contrast, *L. plantarum* is highly versatile and found in many different ecological niches such as vegetables, meat, fish, and dairy products [4-10] as well as in the gastro-intestinal tract [11-13]. *Lactobacillus plantarum* is a facultative heterofermentative organism that is closely related to *Lactobacillus paraplantarum*, *Lactobacillus pentosus* and the recently identified species *Lactobacillus fabifermentans*[14]. In recent years, an extensive molecular and post-genomics tool box has been established for *L. plantarum* and it has become one of the model micro-organisms in LAB research. This review will in particular focus on the genomic and metabolic diversity of *L. plantarum*. We aim to illustrate that the natural genomic architecture and the metabolic consequences hereof are central to the success of *L. plantarum* in industrial applications and resemble metabolic engineering strategies applied in synthetic biology.

### Lactobacillus plantarum diversity

Already in the pre-genomic era it was recognized that the phenotypic diversity within the *L. plantarum* group is very high. A recent phenotypic characterization of 185 isolates from diverse environments showed that isolates from the same food niche or food type phenotypically clustered largely together, but human fecal isolates were scattered throughout different food clusters, suggesting that they generally originate from the food eaten by the individuals [15].

The genetic diversity was initially catalogued by applying molecular approaches including AFLP and RAPD [13,16,17]. This work was important in establishing molecular markers to discriminate *L. plantarum* from *L. paraplantarum* and *L. pentosus*, which exhibit highly similar carbohydrate utilization properties and cannot be discriminated by 16S rRNA gene sequence analysis. A multilocus sequence-typing (MLST) scheme for this organism was reported which exploits the genetic variation present in six loci of housekeeping genes and can be applied as a molecular tool for identification at the strain level [18](Table 1). Such molecular approaches also revealed the existence of the subspecies *L. plantarum* subsp. *argentoratensis*, which is most frequently found in fermented plant substrates and can be discriminated in a nested PCR approach [12]. A recent diversity study through comparative genome hybridization with DNA microarrays designed on basis of the genome of *L. plantarum* WCFS1 confirmed that strains belonging to this subspecies share distinct genomic features and lack two putative extracellular enzyme complexes predicted to be involved in carbohydrate utilization [15].

**Table 1:** Properties of *L. plantarum* isolates determined by MLST.

					Allele no. at locus:						Source of isolate		
					
Strain no.	Strain	RT	ITS	ST	***pgm***	***ddl***	***gyrB***	***purK1***	***gdh***	***mutS***	Origin	Country	Year
1	WCFS1	ND	ND	1	1	1	1	1	1	1	Human saliva	UK	1956
2	CECT 220 (ATCC 8014)	1	1	2	1	3	1	2	6	5	Corn silage	ND	1948
3	CECT 221 (ATCC 14431)	2	1	3	1	1	6	3	8	1	Grass silage	ND	1960
4	CECT 223	2	1	4	1	5	3	4	7	1	ND	Pamplona, Spain	1987
5	CECT 224	2	1	4	1	5	3	4	7	1	ND	Pamplona, Spain	1987
6	CECT 748^T^ (ATCC 14917)	2	1	5	3	2	1	2	1	1	Pickled cabbage	Denmark	1919
7	CECT 749 (ATCC 10241)	2	1	6	3	2	1	2	3	1	Pickled cabbage	ND	1955
8	CECT 4185 (NCBF 1193)	2	1	7	3	1	2	1	5	1	Silage	ND	1958
9	CECT 4645 (NCBF 965)	4	1	8	2	4	7	8	10	4	Cheese	ND	1958
10	RM28	2	1	9	1	2	1	7	7	3	Wine	Valladolid, Spain	2000
11	RM35	4	1	10	3	2	1	2	2	8	Wine	Toledo, Spain	1998
12	RM38	2	1	11	3	1	4	2	4	2	Wine	Toledo, Spain	1998
13	RM40	3	1	12	1	1	1	5	9	1	Wine	Toledo, Spain	1998
14	RM71	2	1	13	3	1	5	6	5	6	Wine	Valladolid, Spain	2001
15	RM72	2	1	13	3	1	5	6	5	6	Wine	Valladolid, Spain	2001
16	RM73	2	1	14	3	2	1	2	1	7	Wine	Madrid, Spain	2000

RT, Ribotype; ITS, 16S–23S ISR type; ST, sequence type; ND, no data available.

Reprinted from [18] with permission from the Society for General Microbiology Ltd.

### Applications of Lactobacillus plantarum

In line with its ability to grow and operate in many different niches, *L. plantarum* is important for different food and health applications. It is a ubiqitious and often one of the dominant species in foods such as sauerkraut, pickles, olives, sourdough and kimchi [19]**.** In many of these fermentations *L. plantarum* dominates especially in the later stages of fermentation, presumably because of its high acid tolerance [20,21]. Over the past decade, several groups have focused on the role of *L. plantarum* in sourdough fermentations, making it one of the best characterized vegetal substrate fermentation processes, as reviewed in depth recently [22]. Meta-transcriptome analysis of sourdough fermentations with DNA microarrays has allowed the global analysis of community dynamics in sourdough fermentations beyond pure populations [23-25]. In these fermentations, *L. plantarum* appears to be subjected to catabolite repression indicating that it is unlikely to play a major role in the utilization of maltose. Other groups have highlighted the importance of intra- and inter-species communication with a specific focus on communication between *L. plantarum* and *L. sanfranciscencis* strains. Quorum sensing communication involving *plnA*- and *luxS*-dependent pathways invoked multiple regulatory responses that directly influence community dynamics as well as the activity of community members [26].

*L. plantarum* is also applied as a probiotic. Over the last decade there have been a growing number of studies aimed at deciphering the potential beneficial effects of *L. plantarum* strains on human health [27]. Strain *L plantarum* 299v is marketed as a probiotic and a number of clinical intervention studies have been published, as reviewed by [28,29]. Interestingly, transcriptome analysis using ileal and colonic biopsies from human intervention studies with this strain revealed that it specifically adapts its metabolic capacity in the human intestine for carbohydrate acquisition and expression of exopolysaccharide and proteinaceous cell-surface compounds [30]. In return, interventions with strain *L. plantarum* WCFS1 were shown to exert distinct and reproducible transcriptional responses in duodenal mucosal biopsies. Consumption of live *L. plantarum* bacteria in different growth phases revealed striking differences in modulation of NF-κB-dependent pathways [31]. These observations shed new light on the molecular cross-talk between ingested *L. plantarum* and the host, although the relevance to host health remains to be established.

### Genome sequencing

Detailed molecular and transcriptomics studies are only possible when the genome sequences are available. The genome of *L. plantarum* strain WCFS1, a single colony isolate of strain NCIMB8826 that was originally isolated from human saliva, was the first to be fully sequenced, and it was in fact the first of any *Lactobacillus* genomes to be published [32]. It consists of a 3.3 Mb chromosome, still the largest of any sequenced lactic acid bacteria to date, and three plasmids of 1.9 kb, 2.3 kb and 36.1 kb (Table 2). A variety of bioinformatics tools has since been used to predict function of genes and gene clusters [33-35], to reconstruct metabolic pathways [36-39], to reconstruct regulatory networks [40-42], to compare with genomes of other lactic acid bacteria [43-45], and to store and visualize these results in a user-friendly way [46]. The genome of the original WCFS1 strain has recently been resequenced using Illumina technology, revealing nearly 100 SNPs and indels, and fully re-annotated (Siezen, Francke, Boekhorst, Renckens, Kleerebezem, van Hijum, unpublished data). This small number (0.003%) of nucleotide corrections detected using modern high-throughput Illumina sequencing technology emphasizes that the original sequencing 10 years ago using Sanger technology was very thorough [32].

**Table 2:** Summary of sequenced genomes of *Lactobacillus plantarum* strains

	WCFS1	JDM1	ST-III	ATCC 14917	NC8	KCA1
**Genome size (bp)**	3,308,274	3,197,759	3,254,376	~3.2 Mb	~3.2 Mb	~3.4 Mb
**contigs**	1	1	1	36	67	84
**GC%**	44.5	44.7	44.6	44.0	45.0	45.3
**CDS**	3007	2948	2996	3154	~3000	~3200
**plasmids**	3	2	1	?	?	0
**Accession code**	NC_004567	CP001617	NC_014554	NZ_ACGZ02000000		
**source**	human saliva	grass silage?	kimchi	pickled cabbage	grass silage	human vagina
**reference**	[32]	[50]	[51]	Human Genome Sequencing Center, Baylor College of Medicine, Houston, Texas, USA	Axelsson et al, unpublished	Anukam et al, unpublished

The flexible and adaptive behaviour of *L. plantarum* was first found to be reflected in the chromosome of strain WCFS1, which encodes a large variety of proteins involved in sugar uptake and utilization, and accompanying regulatory proteins [32]. This allows the organisms to grow on numerous carbon sources as it includes 25 complete PTS enzyme II complexes, several incomplete PTS complexes, and another 30 transport systems predicted to transport various carbohydrates; the genes encoding transporters are usually in gene cassettes clustered with genes encoding enzymes and regulatory proteins involved in sugar metabolism.

The chromosome also encodes over 200 putative extracellular proteins, most of which should be displayed at the cell surface, as they are predicted to be bound to cell-envelope components in several ways [33,35,44,45]. Some of these extracellular proteins are also encoded in specific gene cassettes (e.g. the *csc* genes [33]), and their primary occurrence in plant-associated gram-positive bacteria suggests a possible role in degradation and utilization of plant oligo- or poly-saccharides. This large number of surface-bound extracellular proteins is also likely to contribute to the large flexibility in interactions with its environment.

 It was hypothesized that the *L. plantarum* WCFS1 chromosome contains specific regions that are dedicated to interactions with the environment, designated life-style adaptation regions. These regions are clustered near the origin of replication, exemplified by the region between 3.0 and 3.3 Mb which includes a large proportion of the sugar utilization cassettes as well as genes encoding extracellular functions [32]. This entire region has a lower GC content (41.5% vs 44.5% for the whole chromosome), and many of these genes display deviation of nucleotide composition, consistent with a foreign origin. Thus, based on this single genome sequence, it was suggested that *L. plantarum* has lifestyle adaptation regions that could be “used to effectively adapt to the changes in conditions encountered in the numerous environmental niches in which this microbe is found” [32].

### Genome diversity analysis based on whole genomes by comparative genome hybridization (CGH)

**Figure 1:**
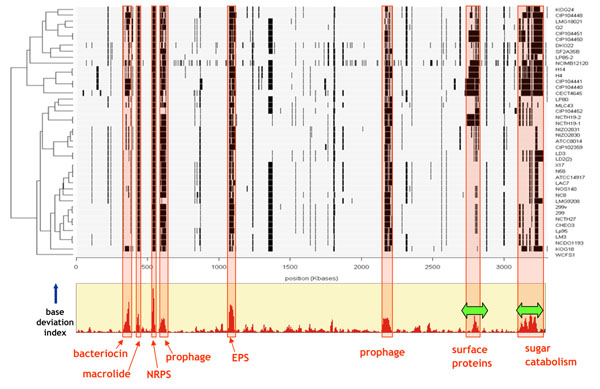
**Comparative genome hybridization (CGH) analysis of *L. plantarum* isolates.** The genes are ordered based on their location in the genome sequence of *L. plantarum* WCFS1 (horizontal axis). Black bars indicate “absent” genes/regions. Red boxes indicate regions where large differences in presence/absence of genes correspond with a high base-deviation index. Adapted from Figure 2 of [15], with permission from the Society for Applied Microbiology and Blackwell Publishing Ltd.

The genomic diversity of *L. plantarum* on a full genome scale was analyzed by CGH in two separate studies [15,47]. The presence or absence of genes relative to the reference strain WCFS1, of which DNA was spotted on microarrays, was assessed by hybridization of DNA from 19 [47] and 41 other *L. plantarum* strains [15] isolated from a large variety of environmental niches, ranging from fermented milk, vegetable, fruit and meat products to human isolates (intestine, saliva, faeces, spinal fluid, urine, teeth). In the first study [47], the probes on the microarray consisted of a subset of genomic fragments amplified by PCR from the random insert library used for initial sequencing of the *L. plantarum* WCFS1 genome; this microarray covered only 81% of all bases of the WCFS1 genome. The presence or absence of genomic fragments (and encoded genes) in the 19 query strains corresponding to the clones on the array was inferred from a statistical model. In the second study [15], an ORF-based DNA-microarray of *L. plantarum* WCFS1 was used, in which most ORFs were represented by at least three specific oligomer probes, evenly distributed over the gene sequence. This allowed a higher coverage of the genome content and higher resolution analysis of individual gene content in the genomes of 41 other *L. plantarum* strains. It must be stressed that this CGH analysis could only detect presence or absence of genes relative to the single reference genome of strain WCFS1, and did not provide information on additional genes not present in the reference strain, nor did it allow conclusions about the chromosomal location of genes (i.e. gene order). These CGH genotyping results can be displayed as “bar plots” with the chromosomal organization of strain WCFS1 as template, in which a black bar indicates the absence of a gene in a specific strain (Fig. 1). Based on their hybridizations profiles, a distance matrix representing fractional genotype similarity between strains can be constructed, and shown in a hierarchical tree. These bar plots clearly show hot spots with high variability amongst strains, and many of these hot spots, but not all, correspond to regions of high base-deviation index, suggestive of horizontal gene transfer.

All tested *L. plantarum* strains were predicted to lack 9-20% of the genes of the reference genome *L. plantarum* WCFS1, and about 50 genes appeared to be specific for strain WCFS1, as they were not found in any other strain [15]. The predicted absence of genes appeared to occur mainly as functional gene clusters, or cassettes, often organized in operons [15,47]. These cassettes encode known functions such as i) prophages, ii) restriction-modification, iii) exopolysaccharide biosynthesis, iv) bacteriocin and non-ribosomal peptide biosynthesis, and v) carbohydrate utilization. Three large cassettes, encoding macrolide biosynthesis, non-ribosomal peptide biosynthesis (NRPS) and exopolysaccharide biosynthesis (EPS), were only found in strain WCFS1 and have a distinctly lower GC content; presumably they have been acquired by recent horizontal gene cassette transfer.

**Figure 2:**
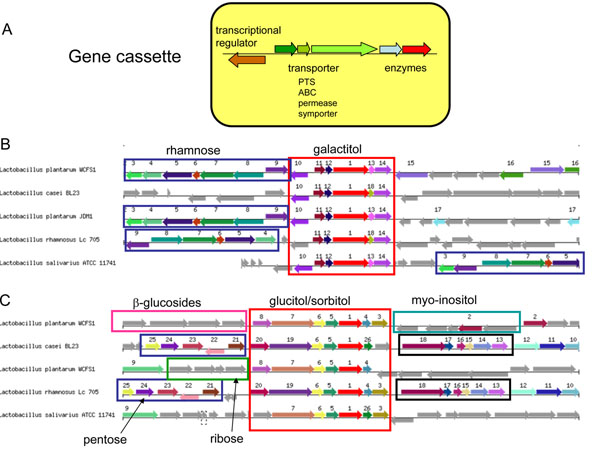
**Gene cassettes for sugar utilization.** (A) General structure of a functional gene cassette; (B) example of rhamnose and galactitol utilization cassettes and their organization in two *L. plantarum* strains and other lactobacilli; (C) example of various sugar utilization cassettes and their organization in various lactobacilli. Orthologs in different strains/species are indicated by corresponding numbers and colors.

Particularly apparent was that the proposed lifestyle adaptation regions with high density of encoded surface proteins and sugar utilization proteins, initially predicted from only the single genome of strain WCFS1 [32], were indeed found to be highly variable in other strains (green arrows in Fig. 1), supporting the hypothesis that life-style adaptation is focused in these regions. A closer inspection of the variable sugar life-style region on the chromosome (from 3.07-3.28 Mb, genes lp_3468- lp_3657) shows that there are many consecutive cassettes of 3-10 genes which are predicted to be involved in utilization of different sugars (Additional file 1) These sugar utilization cassettes usually represent complete functional units, encoding a transporter (permease, PTS or ABC-type), a regulator and enzymes for metabolizing the sugar (Figure 2A). Most cassettes are not unique to *L. plantarum*, but can be found in various other lactobacilli (Figures 2B, 2C) or other bacteria (data not shown). The huge variability of these functional cassettes confirms the enormous flexibility of *L. plantarum* to adapt to different environments and growth substrates.

### Gene-trait matching

A web-tool – PhenoLink – has been developed that facilitates associating bacterial phenotypes to ~omics data (J. Bayjanov, D. Molenaar, R.J. Siezen, S.A.F.T. van Hijum, submitted for publication). This tool uses a Random Forest algorithm [48,49] which builds an ensemble of decision trees to classify huge data sets. This classification method allowed identification of correlations between genotypes (i.e. presence/absence of genes based on CGH) and phenotypes (i.e. growth on carbohydrates, NO_2_ production and stress tolerance) of these 42 *Lactobacillus plantarum* strains (J. Bayjanov, D. Molenaar, R.J. Siezen, S.A.F.T. van Hijum, submitted for publication).

**Figure 3:**
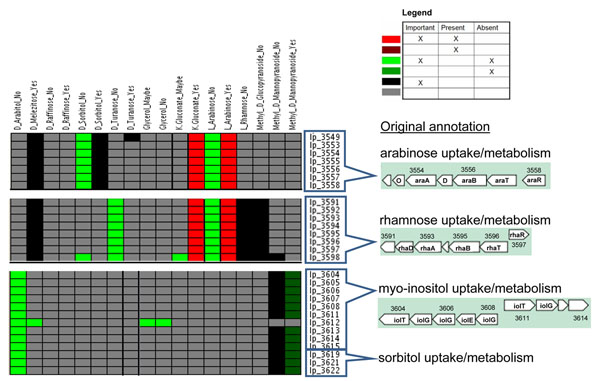
**Genotype-phenotype linkage analysis by Random Forest classification.** Growth (Yes) or non-growth (No) of 42 different *L. plantarum* strains on a variety of sugars was measured. Legend: the color coding used for integration of significance of genes for a certain phenotype with their presence/absence patterns for the different strains. A gene that is found to be important to distinguish strains of different phenotypes is assumed as important. Present (for the majority of strains): gene is present in at least *p* percent (default of 75%) of strains for a given phenotype. Absent (for the majority of strains): gene is absent in at least 75% of strains of a given phenotype. Examples are given of gene cassettes (using gene numbering of strain WCFS1) that are found to be important for phenotype classification of strains.

Examples of preliminary correlations found for some gene cassettes involved in sugar utilization are shown in Figure 3 (courtesy of J. Bayjanov). The 42 strains were tested for growth or no growth on a variety of sugars [15] and the Random Forest algorithm was used to detect correlations with presence or absence of gene clusters. Four gene cassettes are shown which were originally annotated with functions involved in uptake and metabolism of arabinose, rhamnose, myo-inositol and sorbitol [32]. The first cassette (lp_3549-3558) indeed perfectly correlates with the growth on arabinose phenotype, i.e. the gene cluster is present in strains that grow, and absent in strains that do not grow on L-arabinose. However, this gene cluster also correlates with growth on D-sorbitol and K-gluconate, suggesting that these genes may also be involved in metabolizing alternative sugars. The presence of the rhamnose gene cassette (lp_3591-3598) also correlates with growth on L-arabinose and K-gluconate, suggesting that these genes could also be involved in growth on these sugars. Finally, the putative gene clusters for myo-inositol (lp_3604-3615) and sorbitol (lp_3619-3622) utilization are found to correlate with growth on D-arabitol (and not D-sorbitol), suggesting their annotation is too specific and that these systems may be involved in metabolism of various different sugar alcohols.

A preliminary conclusion from this gene-trait matching is that it is not easy to find correlations between phenotypes and genotypes, even with sophisticated algorithms, as both the phenotype and genotype data are inherently noisy. Moreover, classifying genes as present/absent using CGH data based on a single reference genome clearly has its limitations. As shown below, the recently sequenced genomes of other *L. plantarum* strains now provide clues as to why this is the case. On the other hand, the Random Forest methods are able to provide many new leads for the putative function of genes and gene clusters that can be tested experimentally. The examples above already suggest that multiple phenotypes can be linked to a single gene cluster, and vice versa.

### Comparative genomics of 6 sequenced *Lactobacillus plantarum* strains

Complete genome sequences have now been published for *L. plantarum* strains WCFS1 [32], JDM1 [50] and ST-III [51], and the draft genome sequence of type strain *L. plantarum* ATCC14917 is available in Genbank (accession code NZ_ACGZ02000000). Two additional draft genome sequences are available from strains NC8 and KCA1 (Table 2). An extensive comparative analysis of these six genomes has been performed, providing detailed insight into core genes, variable or accessory genes and gene cassettes, genome synteny, transposable elements, and functional adaptation to growth on various substrates (Siezen, Anukam, Axelsson, Francke, Boekhorst, Renckens, Kleerebezem, van Hijum, unpublished data). Some preliminary conclusions and examples will be presented below.

### Comparison of CGH vs. genome sequences

Three strains of the six sequenced genomes were previously included in the CGH analysis, i.e. strains WCFS1, ATCC14917 and NC8 [15]. This allowed a validation of the accuracy of the CGH analysis. The full genome sequences show that several genes are actually present that were classified as absent by CGH (false negatives); the majority of these are in the highly variable gene clusters for prophages, plantaricin and EPS biosynthesis (see below). The main reason for missing these genes by CGH is that the percentage identity of nucleotide sequence for certain genes or even gene cassettes is too low for hybridization on the microarray (Siezen, Anukam, Axelsson, Francke, Boekhorst, Renckens, Kleerebezem, van Hijum, unpublished data). The cut-off for reliable gene detection by hybridization appears to be at an overall 80-90% nucleotide sequence identity of genes, but this will also depend on the choice of probe positions (three 60-mer probes were used for most genes in the CGH experiment [15]).

**Figure 4:**
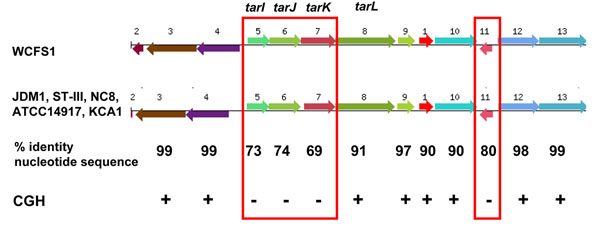
**Diversity of *tar* genes for wall teichoic acid biosynthesis.** Genome organization surrounding the ribitol-type teichoic acid biosynthesis genes *tarIJKL* in *L. plantarum* strains. The percentage nucleotide sequence identity is shown between reference strain WCFS1 and the other 5 strains (which themselves are nearly identical). CGH indicates whether these genes were identified by CGH in strains NC8 and ATCC14917; red boxes signify genes that were not identified by CGH. *tarI:* D-ribitol-5-phosphate cytidylyltransferase; *tarJ*: ribitol-5-phosphate 2-dehydrogenase; *tarK*: ribitol-phosphotransferase; *tarL*: ribitol-phosphotransferase.

An example of genes missed by CGH is the *tarIJKL* gene cluster, encoding wall teichoic acid biosynthesis proteins. Tomita et al [52] sequenced the teichoic acid biosynthesis genes (clusters of 3 and 4 genes) in 18 *L. plantarum* strains, compared these with strain WCFS1 and found two sequence variants which correlate with glycerol-type and ribitol-type teichoic acids. The full genomes now show that these *tar* genes are nearly identical in strains JDM1, ATCC14917, ST-III, NC8 and KCA1, but show only 69-74% nucleotide sequence identity to the *tarIJK* genes of strain WCFS1 (see Figure 4), and hence were missed by the CGH analysis using WCFS1 as the reference.

### Diversity of gene cassettes

In general, there is very high conservation of gene order (synteny) in the six sequenced chromosomes of the *L. plantarum* strains, and in sequence identity of orthologs. However, there are several highly variable regions in the chromosome which deviate from this rule, and some examples are given below.

#### Highly variable cassettes

**Figure 5:**
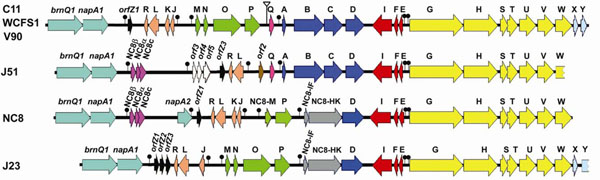
**Diversity of plantaricin (*pln*) gene clusters.** Mosaic *pln* loci from strains of *Lactobacillus plantarum*. Lollipops indicate regulated promoters. The genes *brnQ1* and *napA1* at the upper end of the *pln* loci are not part of the *pln* regulon but they are included in the gene map to signify the upper boundary of the *pln* locus. Note that the lower ends of the *pln* loci of J51, NC8 and J23 are not completely sequenced. Reproduced from [55], with permission from Elsevier Inc.

**Figure 6:**
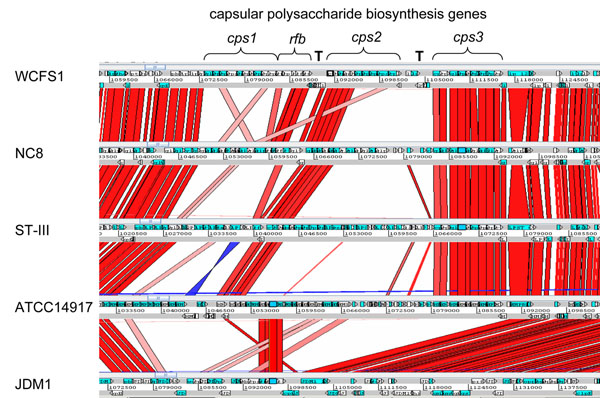
**Diversity of capsular polysaccharide biosynthesis genes.** Comparison of genome organization surrounding the large cluster of *cps* biosynthesis genes. Genes are represented by blue and white arrows, in forward and reverse strands. Shades of connecting bars indicate high sequence identity (bright red) to low sequence identity (pink). The blue connecting bar indicates a reverse orientation. The 3 consecutive *cps* gene clusters in WCFS1 are indicated, and the *rfbACBD* genes. T indicates transposases of IS elements. Picture drawn with Artemis Comparison Tool (ACT)[66].

- Prophages, IS elements and transposases: As expected, the cassettes encoding prophages and transposases (of IS elements) are highly variable, both in gene content and in position of insertion in the chromosomes. Details will be described in (Siezen, Anukam, Axelsson, Francke, Boekhorst, Renckens, Kleerebezem, van Hijum, unpublished data).

- Plantaricin biosynthesis genes. The plantaricin (*pln*) gene cluster of *L. plantarum* of about 25 genes (Figure 5), encodes the biosynthesis of various class IIb bacteriocins, whose full activity depends on the action of two different peptides [53,54]. The *pln* gene cluster has been previously sequenced from six *L. plantarum* strains (including WCFS1 and NC8) and found to be a highly variable and mosaic region, with parts being relatively conserved and other parts less conserved [55,56]. A PCR analysis directed to 27 genes of this *pln* cluster in 33 *L. plantarum* strains of oenological origin found even more variation and led to a subdivision into seven groups, named plantaritypes [56]. The six fully sequenced genomes (Table 2) show the same high variability, but also demonstrate that the conservation and synteny of genes flanking the *pln* locus is very high (Siezen, Anukam, Axelsson, Francke, Boekhorst, Renckens, Kleerebezem, van Hijum, unpublished data).

- CPS/EPS biosynthesis genes. The chromosome of strain WCFS1 has 3 consecutive *cps* clusters (*cps1*, *cps2*, *cps3*), separated by transposase genes (and their fragments), encoding proteins involved in biosynthesis and export of extracellular or capsular polysaccharides. At the same position in the chromosome, the other 5 sequenced genomes also have *cps* clusters (Figure 6). Some parts of these clusters are shared, but many parts contain completely different *cps* genes. The *cps1* cluster of strain WCFS1 is not present in any of the other sequenced genomes, while the *cps3* cluster and parts of the *cps2* cluster are shared by 4 strains, but lacking in JDM1. This high variability presumably leads to variation in the structure of capsular and exopolysaccharides. A similar variability of EPS gene cassettes has been observed in other LAB [57-62].

#### Life-style cassettes

The enormous variability of cassettes in the sugar life-style region deduced from CGH analysis (Additionl file 1) [15,47] led to the hypothesis “…. that similar lifestyle adaptation islands will exist in other strains of *L. plantarum*. This would also imply that these strains contain a high number of genes with related functions accumulated within their lifestyle adaptation region that are absent from the WCFS1 genome.” [47].

**Figure 7:**
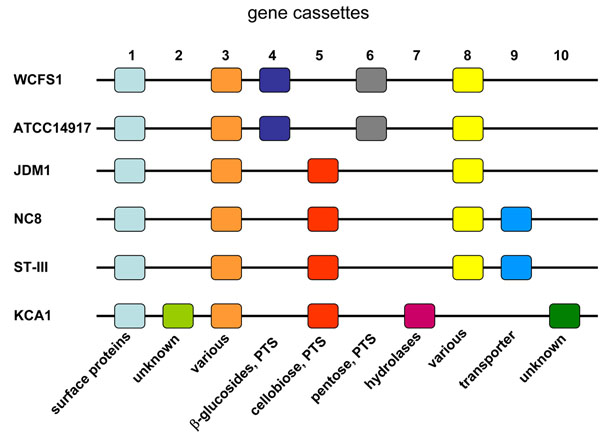
**Modularity and variability of life-style gene cassettes.** Life-style region corresponding to genes lp_3114-lp_3150 (from 2.78-2.82 Mb on the chromosome) in strain WCFS1, represented as gene cassettes. Each cassette contains 3-18 genes (see example in Figure 2A); identical colors indicate corresponding cassettes in other strains. Putative functions of proteins encoded in the cassettes are indicated.

Comparison of the 6 sequenced genomes now shows that indeed many novel cassettes are present in these variable regions that are absent in strain WCFS1 (Siezen, Anukam, Axelsson, Francke, Boekhorst, Renckens, Kleerebezem,van Hijum, unpublished data). A typical example is shown in Figure 7, representing the diversity of cassettes in a small part of the life-style region corresponding to genes lp_3114-lp_3150 (from 2.78-2.82 Mb on the chromosome) in strain WCFS1. In addition to the five known cassettes present in strain WCFS1, another five novel cassettes are found in the other sequenced strains, and these would not have been detected in the original CGH analysis using only WCFS1 as a reference. Strains WCFS1 and ATCC14917 share the same 5 cassettes, and strains NC8 and ST-III share a different set of 5 cassettes, while 3 cassettes are unique to strain KCA1. Cassette 5 encoding putative cellobiose utilization appears to replace cassette 4 encoding β-glucosides utilization at the same position in the chromosome.

### Diversity at single gene/protein level

Diversity between strains is even seen within *L. plantarum* genes and proteins at the level of numbers of repeated domains or motifs, particularly in extracellular proteins, again suggesting variability between strains in interactions with their environment.

**Figure 8:**
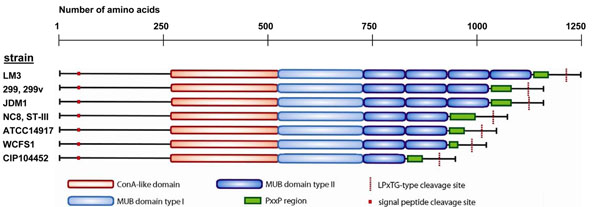
**Diversity of domain composition within the mannose-specific adhesion (Msa).** Determined by sequencing of the *msa* gene in a subset of *L. plantarum* strains, highlighting the variation in numbers of mucus-binding (MUB) domains and PxxP-repeats. Adapted from [63], with permission from Wageningen Academic Publishers, the Netherlands.

One published example is an extracellular, peptidoglycan-bound mannose-specific adhesin (*msa* gene; lp_1229 in strain WCFS1) which has variations in the number of mucus-binding (Mub) domains and PxxP spacer motifs in different strains, which could relate to differences in mucus-binding efficiency [63](Figure 8). Another example is a very large extracellular, membrane-anchored protein (encoded by lp_1303a in strain WCFS1) of unknown function that has a middle domain with hundreds of repeats of SD (Ser-Asp); possibly the Ser residues are glycosylated by glycosyl transferases [32], as three adjacent genes in the *L. plantarum* genomes encode putative glycosyl transferases. The N-terminal domain (includes signal peptide) and C-terminal domain (includes membrane anchor) are nearly identical in this protein from all sequenced *L. plantarum* strains, but there is a large variation in the number of SD repeats ranging from 242 repeats in strain JDM1 to 801 repeats in strain WCFS1.

## Conclusions

Genome sequencing and comparative genomics of *L. plantarum* has revealed a high genomic diversity, versatility and flexibility which are at the heart of its success in diverse niches and applications. One of the most striking observations is the occurrence of genomic islands harboring mosaic modules or cassettes of carbohydrate utilization genes, likely acquired through horizontal gene transfer. Many of these cassettes are also found in other LAB (see for instance Figure 2) and other gram-positive bacteria. *L. plantarum* seems to be a master in acquiring and shuffling these cassettes, but it cannot be excluded that this shuffling propensity is also linked with other gram-positive bacteria. By its ability to acquire and assemble gene cassettes required for the utilization of carbohydrates, *L. plantarum* seems to have developed a “natural metabolic engineering approach” that allows it to optimize its genome for growth in specific niches, especially those rich in plant carbohydrates. In that aspect, there are interesting synergies with the approaches applied nowadays in metabolic engineering for biofuels production. The cellular traits and catabolic capacities that are sought when constructing biofuels-producing *S. cerevisiae* and *E. coli* are prospected in nature, introduced into the host and subsequently optimized through advanced molecular and evolutionary engineering strategies [64].

It seems likely that in *L. plantarum* there could be an important role in this respect for diversity generating molecular structures such as (conjugative) plasmids, IS elements and transposons. To date, experimental data showing that transfer of such elements is an effective means to increase the catabolic potential of lactic acid bacteria are scarce. Recently, it was shown that conjugative transfer of transposon Tn6098 encoding alpha-galactosides metabolism in *Lactococcus lactis* enable a dairy isolate to grow in soy milk, a substrate rich in α-galactosides [65]. On the other hand, the numerous cassettes in *L. plantarum* appear to be directly linked to each other, with no evidence of flanking or intervening elements from plasmids or IS elements. Further investigation into the molecular mechanisms by which *L. plantarum* is able to acquire and assemble such gene cassettes encoding functional pathways into its chromosome should therefore be of importance to learn how to harness this natural metabolic engineer.

## Competing interests

The authors declare they have no competing interests.

## Authors’contributions

RJS conceived the review and gathered, restructured and analyzed data. RJS and JHV jointly drafted and wrote the manuscript.

## List of abbreviations used

ABC: ATP-binding cassette; ACT: Artemis comparison tool; AFLP: amplified fragment-length polymorphism; CGH: comparative genome hybridization; CPS: capsular polysaccharide; EPS: extracellular polysaccharide; GC: guanine & cytosine; LAB: lactic acid bacteria; IS: insertion sequence; MLST: multi-locus sequence typing; ORF: open-reading frame; PCR: polymerase chain reaction; PTS: phospho-transferase system; RAPD: random amplification of polymorphic DNA; SNP: single nucleotide polymorphism.

## Supplementary Material

Additional file 1 Diversity in presence/absence of gene clusters in 42 *L. plantarum* strains in part of the sugar life-style island region, according to CGH analysis. Click here for file

## References

[B1] Felis GE Dellaglio F Taxonomy of Lactobacilli and Bifidobacteria Curr Issues Intest Microbiol 2007 8 2 44 61 17542335

[B2] Siezen RJ Wilson G Probiotics genomics Microb Biotechnol 2010 3 1 1 9 10.1111/j.1751-7915.2009.00159.x 21255300 PMC3815941

[B3] Macklaim JM Gloor GB Anukam KC Cribby S Reid G At the crossroads of vaginal health and disease, the genome sequence of *Lactobacillus iners* AB-1 Proc Natl Acad Sci U S A 2011 108 Suppl 1 4688 4695 21059957 10.1073/pnas.1000086107 PMC3063587

[B4] Gardner NJ Savard T Obermeier P Caldwell G Champagne CP Selection and characterization of mixed starter cultures for lactic acid fermentation of carrot, cabbage, beet and onion vegetable mixtures Int J Food Microbiol 2001 64 3 261 275 10.1016/S0168-1605(00)00461-X 11294348

[B5] Ercolini D Hill PJ Dodd CE Bacterial community structure and location in Stilton cheese Appl Environ Microbiol 2003 69 6 3540 3548 10.1128/AEM.69.6.3540-3548.2003 12788761 PMC161494

[B6] Aryanta RW Fleet GH Buckle KA The occurrence and growth of microorganisms during the fermentation of fish sausage Int J Food Microbiol 1991 13 2 143 155 10.1016/0168-1605(91)90056-U 1909546

[B7] Mundt JO Hammer JL Lactobacilli on plants Appl Microbiol 1968 16 9 1326 1330 5676407 10.1128/am.16.9.1326-1330.1968 PMC547649

[B8] Aquilanti L Santarelli S Silvestri G Osimani A Petruzzelli A Clementi F The microbial ecology of a typical Italian salami during its natural fermentation Int J Food Microbiol 2007 120 1-2 136 145 10.1016/j.ijfoodmicro.2007.06.010 17628130

[B9] Ganzle MG Vermeulen N Vogel RF Carbohydrate, peptide and lipid metabolism of lactic acid bacteria in sourdough Food Microbiol 2007 24 2 128 138 10.1016/j.fm.2006.07.006 17008155

[B10] Aymerich T Martin B Garriga M Hugas M Microbial quality and direct PCR identification of lactic acid bacteria and nonpathogenic staphylococci from artisanal low-acid sausages Appl Environ Microbiol 2003 69 8 4583 4594 10.1128/AEM.69.8.4583-4594.2003 12902246 PMC169148

[B11] Ahrne S Nobaek S Jeppsson B Adlerberth I Wold AE Molin G The normal *Lactobacillus* flora of healthy human rectal and oral mucosa J Appl Microbiol 1998 85 1 88 94 10.1046/j.1365-2672.1998.00480.x 9721659

[B12] Bringel F Castioni A Olukoya DK Felis GE Torriani S Dellaglio F *Lactobacillus plantarum* subsp. *argentoratensis* subsp. nov., isolated from vegetable matrices Int J Syst Evol Microbiol 2005 55 Pt 4 1629 1634 16014493 10.1099/ijs.0.63333-0

[B13] Bringel F Quenee P Tailliez P Polyphasic investigation of the diversity within *Lactobacillus plantarum* related strains revealed two L. plantarum subgroups Syst Appl Microbiol 2001 24 4 561 571 10.1078/0723-2020-00061 11876364

[B14] De Bruyne K Camu N De Vuyst L Vandamme P *Lactobacillus fabifermentans* sp. nov. and *Lactobacillus cacaonum* sp. nov., isolated from Ghanaian cocoa fermentations Int J Syst Evol Microbiol 2009 59 Pt 1 7 12 19126714 10.1099/ijs.0.001172-0

[B15] Siezen RJ Tzeneva VA Castioni A Wels M Phan HT Rademaker JL Starrenburg MJ Kleerebezem M Molenaar D van Hylckama Vlieg JE Phenotypic and genomic diversity of *Lactobacillus plantarum* strains isolated from various environmental niches Environ Microbiol 2010 12 3 758 773 10.1111/j.1462-2920.2009.02119.x 20002138

[B16] Torriani S Clementi F Vancanneyt M Hoste B Dellaglio F Kersters K Differentiation of *Lactobacillus plantarum*, *L. pentosus* and *L. paraplantarum* species by RAPD-PCR and AFLP Syst Appl Microbiol 2001 24 4 554 560 10.1078/0723-2020-00071 11876363

[B17] Torriani S Felis GE Dellaglio F Differentiation of *Lactobacillus plantarum*, *L. pentosus*, and *L. paraplantarum* by recA gene sequence analysis and multiplex PCR assay with recA gene-derived primers Appl Environ Microbiol 2001 67 8 3450 3454 10.1128/AEM.67.8.3450-3454.2001 11472918 PMC93042

[B18] de Las Rivas B Marcobal A Munoz R Development of a multilocus sequence typing method for analysis of *Lactobacillus plantarum* strains Microbiology 2006 152 Pt 1 85 93 16385118 10.1099/mic.0.28482-0

[B19] Breidt F McFeeters RF Díaz-Muñiz I Doyle MP, Beuchat LR Fermented vegetables Food Microbiology: Fundamentals and Frontiers 2007 3 Washington DC: ASM Press 783 793

[B20] Luxananil P Promchai R Wanasen S Kamdee S Thepkasikul P Plengvidhya V Visessanguan W Valyasevi R Monitoring *Lactobacillus plantarum* BCC 9546 starter culture during fermentation of Nham, a traditional Thai pork sausage Int J Food Microbiol 2009 129 3 312 315 10.1016/j.ijfoodmicro.2008.12.011 19157611

[B21] Plengvidhya V Breidt F Jr. Lu Z Fleming HP DNA fingerprinting of lactic acid bacteria in sauerkraut fermentations Appl Environ Microbiol 2007 73 23 7697 7702 10.1128/AEM.01342-07 17921264 PMC2168044

[B22] De Vuyst L Vranckena G Ravytsa F Rimauxa T Weckx S S. Biodiversity, ecological determinants, and metabolic exploitation of sourdough microbiota Food Microbiology 2009 26 7 666 675 10.1016/j.fm.2009.07.012 19747599

[B23] Weckx S Allemeersch J Van der Meulen R Vrancken G Huys G Vandamme P Van Hummelen P De Vuyst L Development and validation of a species-independent functional gene microarray that targets lactic acid bacteria Appl Environ Microbiol 2009 75 20 6488 6495 10.1128/AEM.01055-09 19684161 PMC2765132

[B24] Weckx S Allemeersch J Van der Meulen R Vrancken G Huys G Vandamme P Van Hummelen P De Vuyst L Metatranscriptome analysis for insight into whole-ecosystem gene expression during spontaneous wheat and spelt sourdough fermentations Appl Environ Microbiol 2011 77 2 618 626 10.1128/AEM.02028-10 21097589 PMC3020550

[B25] Weckx S Van der Meulen R Allemeersch J Huys G Vandamme P Van Hummelen P De Vuyst L Community dynamics of bacteria in sourdough fermentations as revealed by their metatranscriptome Appl Environ Microbiol 2010 76 16 5402 5408 10.1128/AEM.00570-10 20581179 PMC2918950

[B26] Di Cagno R De Angelis M Limitone A Minervini F Simonetti MC Buchin S Gobbetti M Cell-cell communication in sourdough lactic acid bacteria: a proteomic study in *Lactobacillus sanfranciscensis* CB1 Proteomics 2007 7 14 2430 2446 10.1002/pmic.200700143 17623302

[B27] Connelly P Lactobacillus plantarum - A literature review of therapeutic benfefits Journal of the Australian Traditional Medicine Society 2008 14 2 79 82

[B28] Klarin B Molin G Jeppsson B Larsson A Use of the probiotic *Lactobacillus plantarum* 299 to reduce pathogenic bacteria in the oropharynx of intubated patients: a randomised controlled open pilot study Crit Care 2008 12 6 R136 10.1186/cc7109 18990201 PMC2646346

[B29] Gareau MG Sherman PM Walker WA Probiotics and the gut microbiota in intestinal health and disease Nat Rev Gastroenterol Hepatol 2010 7 9 503 514 10.1038/nrgastro.2010.117 20664519 PMC4748966

[B30] Marco ML de Vries MC Wels M Molenaar D Mangell P Ahrne S de Vos WM Vaughan EE Kleerebezem M Convergence in probiotic *Lactobacillus* gut-adaptive responses in humans and mice ISME J 2010 4 11 1481 1484 10.1038/ismej.2010.61 20505752

[B31] van Baarlen P Troost FJ van Hemert S van der Meer C de Vos WM de Groot PJ Hooiveld GJ Brummer RJ Kleerebezem M Differential NF-kappaB pathways induction by *Lactobacillus plantarum* in the duodenum of healthy humans correlating with immune tolerance Proc Natl Acad Sci U S A 2009 106 7 2371 2376 10.1073/pnas.0809919106 19190178 PMC2650163

[B32] Kleerebezem M Boekhorst J van Kranenburg R Molenaar D Kuipers OP Leer R Tarchini R Peters SA Sandbrink HM Fiers MW Complete genome sequence of *Lactobacillus plantarum* WCFS1 Proc Natl Acad Sci U S A 2003 100 4 1990 1995 10.1073/pnas.0337704100 12566566 PMC149946

[B33] Siezen R Boekhorst J Muscariello L Molenaar D Renckens B Kleerebezem M *Lactobacillus plantarum* gene clusters encoding putative cell-surface protein complexes for carbohydrate utilization are conserved in specific gram-positive bacteria BMC Genomics 2006 7 126 10.1186/1471-2164-7-126 16723015 PMC1534035

[B34] Siezen RJ van Enckevort FH Kleerebezem M Teusink B Genome data mining of lactic acid bacteria: the impact of bioinformatics Curr Opin Biotechnol 2004 15 2 105 115 10.1016/j.copbio.2004.02.002 15081047

[B35] Boekhorst J Wels M Kleerebezem M Siezen RJ The predicted secretome of *Lactobacillus plantarum* WCFS1 sheds light on interactions with its environment Microbiology 2006 152 Pt 11 3175 3183 17074889 10.1099/mic.0.29217-0

[B36] Francke C Siezen RJ Teusink B Reconstructing the metabolic network of a bacterium from its genome Trends Microbiol 2005 13 11 550 558 10.1016/j.tim.2005.09.001 16169729

[B37] Teusink B van Enckevort FH Francke C Wiersma A Wegkamp A Smid EJ Siezen RJ In silico reconstruction of the metabolic pathways of *Lactobacillus plantarum*: comparing predictions of nutrient requirements with those from growth experiments Appl Environ Microbiol 2005 71 11 7253 7262 10.1128/AEM.71.11.7253-7262.2005 16269766 PMC1287688

[B38] Teusink B Wiersma A Jacobs L Notebaart RA Smid EJ Understanding the adaptive growth strategy of *Lactobacillus plantarum* by in silico optimisation PLoS Comput Biol 2009 5 6 e1000410 10.1371/journal.pcbi.1000410 19521528 PMC2690837

[B39] Teusink B Wiersma A Molenaar D Francke C de Vos WM Siezen RJ Smid EJ Analysis of growth of *Lactobacillus plantarum* WCFS1 on a complex medium using a genome-scale metabolic model J Biol Chem 2006 281 52 40041 40048 10.1074/jbc.M606263200 17062565

[B40] Francke C Kerkhoven R Wels M Siezen RJ A generic approach to identify Transcription Factor-specific operator motifs; Inferences for LacI-family mediated regulation in *Lactobacillus plantarum* WCFS1 BMC Genomics 2008 9 145 10.1186/1471-2164-9-145 18371204 PMC2329647

[B41] Wels M Francke C Kerkhoven R Kleerebezem M Siezen RJ Predicting cis-acting elements of *Lactobacillus plantarum* by comparative genomics with different taxonomic subgroups Nucleic Acids Res 2006 34 7 1947 1958 10.1093/nar/gkl138 16614445 PMC1435977

[B42] Wels M Overmars L Francke C Kleerebezem M Siezen RJ Reconstruction of the regulatory network of *Lactobacillus plantarum* WCFS1 on basis of correlated gene expression and conserved regulatory motifs Microb Biotechnol 2010 10.1111/j.1751-7915.2010.00217.x PMC3818992 21375715

[B43] Boekhorst J Siezen RJ Zwahlen MC Vilanova D Pridmore RD Mercenier A Kleerebezem M de Vos WM Brussow H Desiere F The complete genomes of *Lactobacillus plantarum* and *Lactobacillus johnsonii* reveal extensive differences in chromosome organization and gene content Microbiology 2004 150 Pt 11 3601 3611 15528649 10.1099/mic.0.27392-0

[B44] Kleerebezem M Hols P Bernard E Rolain T Zhou M Siezen RJ Bron PA The extracellular biology of the lactobacilli FEMS Microbiol Rev 2010 34 2 199 230 10.1111/j.1574-6976.2009.00208.x 20088967

[B45] Zhou M Theunissen D Wels M Siezen RJ LAB-Secretome: a genome-scale comparative analysis of the predicted extracellular and surface-associated proteins of Lactic Acid Bacteria BMC Genomics 2010 11 651 10.1186/1471-2164-11-651 21092245 PMC3017865

[B46] Kerkhoven R van Enckevort FH Boekhorst J Molenaar D Siezen RJ Visualization for genomics: the Microbial Genome Viewer Bioinformatics 2004 20 11 1812 1814 10.1093/bioinformatics/bth159 14988111

[B47] Molenaar D Bringel F Schuren FH de Vos WM Siezen RJ Kleerebezem M Exploring *Lactobacillus plantarum* genome diversity by using microarrays J Bacteriol 2005 187 17 6119 6127 10.1128/JB.187.17.6119-6127.2005 16109953 PMC1196139

[B48] Breiman L Random Forests Machine Learning 2001 45 1 5 32 10.1023/A:1010933404324

[B49] Liaw A Wiener M Classification and regression by random Forest R News 2002 2 3 18 22

[B50] Zhang ZY Liu C Zhu YZ Zhong Y Zhu YQ Zheng HJ Zhao GP Wang SY Guo XK Complete genome sequence of *Lactobacillus plantarum* JDM1 J Bacteriol 2009 191 15 5020 5021 10.1128/JB.00587-09 19465650 PMC2715720

[B51] Wang Y Chen C Ai L Zhou F Zhou Z Wang L Zhang H Chen W Guo B Complete genome sequence of the probiotic *Lactobacillus plantarum* ST-III J Bacteriol 2011 193 1 313 314 10.1128/JB.01159-10 21037001 PMC3019943

[B52] Tomita S Irisawa T Tanaka N Nukada T Satoh E Uchimura T Okada S Comparison of components and synthesis genes of cell wall teichoic acid among *Lactobacillus plantarum* strains Biosci Biotechnol Biochem 2010 74 5 928 933 10.1271/bbb.90736 20460720

[B53] Nissen-Meyer J Oppegard C Rogne P Haugen HS Kristiansen PE Structure and Mode-of-Action of the Two-Peptide (Class-IIb) Bacteriocins Probiotics Antimicrob Proteins 2010 2 1 52 60 10.1007/s12602-009-9021-z 20383320 PMC2850506

[B54] Nissen-Meyer J Rogne P Oppegard C Haugen HS Kristiansen PE Structure-function relationships of the non-lanthionine-containing peptide (class II) bacteriocins produced by gram-positive bacteria Curr Pharm Biotechnol 2009 10 1 19 37 10.2174/138920109787048661 19149588

[B55] Diep DB Straume D Kjos M Torres C Nes IF An overview of the mosaic bacteriocin pln loci from *Lactobacillus plantarum* Peptides 2009 30 8 1562 1574 10.1016/j.peptides.2009.05.014 19465075

[B56] Saenz Y Rojo-Bezares B Navarro L Diez L Somalo S Zarazaga M Ruiz-Larrea F Torres C Genetic diversity of the pln locus among oenological *Lactobacillus plantarum* strains Int J Food Microbiol 2009 134 3 176 183 10.1016/j.ijfoodmicro.2009.06.004 19604592

[B57] Bourgoin F Pluvinet A Gintz B Decaris B Guedon G Are horizontal transfers involved in the evolution of the *Streptococcus thermophilus* exopolysaccharide synthesis loci? Gene 1999 233 1-2 151 161 10.1016/S0378-1119(99)00144-4 10375631

[B58] Broadbent JR McMahon DJ Welker DL Oberg CJ Moineau S Biochemistry, genetics, and applications of exopolysaccharide production in *Streptococcus thermophilus*: a review J Dairy Sci 2003 86 2 407 423 10.3168/jds.S0022-0302(03)73619-4 12647947

[B59] Rasmussen TB Danielsen M Valina O Garrigues C Johansen E Pedersen MB *Streptococcus thermophilus* core genome: comparative genome hybridization study of 47 strains Appl Environ Microbiol 2008 74 15 4703 4710 10.1128/AEM.00132-08 18539806 PMC2519362

[B60] Berger B Pridmore RD Barretto C Delmas-Julien F Schreiber K Arigoni F Brussow H Similarity and differences in the *Lactobacillus acidophilus* group identified by polyphasic analysis and comparative genomics J Bacteriol 2007 189 4 1311 1321 10.1128/JB.01393-06 17142402 PMC1797336

[B61] Raftis EJ Salvetti E Torriani S Felis GE O'Toole PW Genomic diversity of *Lactobacillus salivarius* Appl Environ Microbiol 2011 77 3 954 965 10.1128/AEM.01687-10 21131523 PMC3028724

[B62] Siezen RJ Bayjanov JR Felis GE van der Sijde MR Starrenburg M Molenaar D Wels M van Hijum SA van Hylckama Vlieg JE Genome-scale diversity and niche adaptation analysis of *Lactococcus lactis* by comparative genome hybridization using multi-strain arrays Microb Biotechnol 2011 10.1111/j.1751-7915.2011.00247.x PMC3818997 21338475

[B63] Gross G Snel J Boekhorst J Smits MA Kleerebezem M Biodiversity of mannose-specific adhesion in *Lactobacillus plantarum* revisited: strain-specific domain composition of the mannose-adhesin Benefical Microbes 2010 1 1 61 66 10.3920/BM2008.1006 21840797

[B64] Alper H Stephanopoulos G Engineering for biofuels: exploiting innate microbial capacity or importing biosynthetic potential? Nat Rev Microbiol 2009 7 10 715 723 10.1038/nrmicro2186 19756010

[B65] Machielsen R Siezen RJ van Hijum SA van Hylckama Vlieg JE Molecular description and industrial potential of Tn6098 conjugative transfer conferring alpha-galactoside metabolism in *Lactococcus lactis* Appl Environ Microbiol 2011 77 2 555 563 10.1128/AEM.02283-10 21115709 PMC3020558

[B66] Carver T Berriman M Tivey A Patel C Bohme U Barrell BG Parkhill J Rajandream MA Artemis and ACT: viewing, annotating and comparing sequences stored in a relational database Bioinformatics 2008 24 23 2672 2676 10.1093/bioinformatics/btn529 18845581 PMC2606163

